# Identification of Quinazolinone Analogs Targeting CDK5 Kinase Activity and Glioblastoma Cell Proliferation

**DOI:** 10.3389/fchem.2020.00691

**Published:** 2020-08-19

**Authors:** Marion Peyressatre, Dominique Patomo Arama, Arthur Laure, Juan A. González-Vera, Morgan Pellerano, Nicolas Masurier, Vincent Lisowski, May C. Morris

**Affiliations:** Institut des Biomolecules Max Mousseron, UMR 5247, CNRS, Université de Montpellier, UFR des Sciences Pharmaceutiques et Biologiques, Montpellier, France

**Keywords:** CDK5, kinase, conformational biosensor, quinazoline, small molecule inhibitor, fluorescence-based screening

## Abstract

CDK5/p25 kinase plays a major role in neuronal functions, and is hyperactivated in several human cancers including glioblastoma and neurodegenerative pathologies such as Alzheimer's and Parkinson's. CDK5 therefore constitutes an attractive pharmacological target. Since the successful discovery and development of Roscovitine, several ATP-competitive inhibitors of CDK5 and peptide inhibitors of CDK5/p25 interface have been developed. However, these compounds suffer limitations associated with their mechanism of action and nature, thereby calling for alternative targeting strategies. To date, few allosteric inhibitors have been developed for successful targeting of protein kinases. Indeed, although this latter class of inhibitors are believed to be more selective than compounds targeting the active site, they have proven extremely difficult to identify in high throughput screens. By implementing a fluorescent biosensor that discriminates against ATP-pocket binding compounds to screen for allosteric inhibitors that target conformational activation of CDK5, we have identified a novel family of quinazolinones. Characterization of these hits and several of their derivatives revealed their inhibitory potential toward CDK5 kinase activity *in vitro* and to inhibit glioblastoma cell proliferation. The quinazolinone derivatives described in this study are the first small molecules reported to target CDK5 at a site other than the ATP pocket, thereby constituting attractive leads for glioblastoma therapeutics and providing therapeutic perspectives for neurodegenerative diseases. These compounds offer alternatives to conventional ATP-competitive inhibitors or peptides targeting CDK5/p25 interface with the potential of bypassing their limitations.

## Introduction

The serine/threonine kinase CDK5, an unconventional member of the cyclin-dependent kinases (CDK) family, is essentially active in post-mitotic neurons where it is involved in neuronal maturation and migration, axonal guidance and synaptic transmission (Dhavan and Tsai, 2001). CDK5 is tightly regulated by its neurospecific partner p35, which anchors it at the membrane in an inactive form (Lim et al., 2001). Following an influx of calcium, p35 undergoes calpain-mediated cleavage into p25, which is released into the cytoplasm in an active complex with CDK5. CDK5 hyperactivity has been described in several neurodegenerative disorders including Alzheimer's and Parkinson's disease as well as amyotrophic lateral sclerosis (ALS) (Cheung and Ip, 2012). In addition, a growing number of studies describe upregulation or hyperactivation of CDK5 in human cancers, indicating that CDK5 constitutes a relevant target in oncology (Catania et al., 2001; Peyressatre et al., 2015; Pozo and Bibb, 2017). Indeed, CDK5-mediated phosphorylation of phosphatidylinositol 3-kinase enhancer PIKE-A stimulates its GTPase activity, in turn activating nuclear Akt, and mediating growth factor-induced migration and invasion of glioblastoma cells (Liu et al., 2008). CDK5 has also been found to phosphorylate dynamin-related protein 1 (DRP1), thereby stimulating its activity in brain tumor-initiating cells; DRP1 activation correlates with poor prognosis in glioblastoma (Xie et al., 2015). More recently CDK5 has been identified as a valuable predictive marker of tumorigenesis and progression in glioma (Yushan et al., 2015). CDK5 therefore constitutes an attractive pharmacological target.

CDK5 has long been considered a relevant pharmacological target in neurodegenerative diseases (Alzheimer's, Parkinson's, Huntington's, ALS) and has more recently become an attractive drug target for cancer therapy (Cheung and Ip, 2012; Pozo and Bibb, 2017; Shupp et al., 2017). Aberrant expression or hyperactivation of this kinase contributes to tumorigenesis and progression of several tumors including cancer stem cells by stimulating proliferation, migration, angiogenesis, and CDK5 activity regulates the DNA damage pathway, chemotherapy resistance and anti-tumor immunity (Lenjisa et al., 2017; Pozo and Bibb, 2017; Shupp et al., 2017).

A wide variety of ATP-competitive inhibitors of CDK/cyclins have been identified in high throughput screening assays, and/or further developed through structure-guided approaches for anticancer therapeutics (Lapenna and Giordano, 2009; Bruyère and Meijer, 2013; Asghar et al., 2015). Whilst first generation pan-CDK inhibitors such as flavopiridol or R-Roscovitine have shown disappointingly low activity and high toxicity in clinical trials, further efforts have yielded second-generation CDK inhibitors with improved potency and selectivity. Notwithstanding the success of such compounds, they ultimately lead to emergence of resistance, calling for new generations of kinase inhibitors, whose mechanism of action does not involve targeting the ATP pocket (Wu et al., 2016).

Most inhibitors developed to target CDK5, including R-Roscovitine, Purvalanol-A, Dinaciclib and AT7519, bind within its ATP-binding pocket (Peyressatre et al., 2015). Since the mechanism of action of ATP-competitive inhibitors is notorious for its lack of selectivity, resulting in a variety of undesirable secondary effects, alternative strategies have been deployed to identify compounds that target sites other than the ATP-binding pocket or the catalytic site and thereby enable development of novel classes of therapeutics (Cirillo et al., 2011; Abate et al., 2013; Bruyère and Meijer, 2013; Asghar et al., 2015). In particular, several peptides have been designed to target the main interface between CDK5 and its regulatory coactivator p25/p35, with the aim of preventing complex formation. Peptides derived from p25/p35 were successfully developed to target CDK5/p25 with high specificity, thereby downregulating CDK5 hyperactivity and Tau hyperphosphorylation, reducing neurodegeneration and preventing Alzheimer's in mouse models without any toxicity (Chin et al., 1999; Zheng et al., 2002, 2010; Shukla et al., 2013; Sundaram et al., 2013). More recently, CDK5i, a peptide inhibitor derived from the activation loop of CDK5 was patented for its ability to selectively disrupt the interaction between CDK5 and p25/p35 without affecting CDK5 physiological activity (Tsai et al., 2019; patent N° WO/2019/055236). Although such peptides constitute promising therapies, they remain problematic for clinical use due to low cell membrane permeability, solubility and bioavailability. A different strategy has consisted in screening for small molecules that target allosteric sites of the kinase rather than the ATP binding pocket. Combining alanine-scanning calculations for locating binding sites, virtual screening for small molecules, molecular dynamics simulations and a BRET-based screen in budding yeast led to the unexpected identification of Tamoxifen as a p25 binder and inhibitor of CDK5/p25 complex formation (Zhang et al., 2011). Taken together these studies provide encouraging perspectives for development of new drugs targeting protein-protein interactions (PPI) rather than the ATP pocket.

CDK activation is a complex process, which is initiated by the interaction with the cyclin subunit, which induces a major conformational reorganization of the kinase fold, and further proceeds through a series of regulatory phosphorylation/dephosphorylation steps (Jeffrey et al., 1995; Morgan, 1995; Russo et al., 1996; Morris et al., 2002). In particular, a segment known as the activation loop (T-loop) of the CDK undergoes a positional switch to a conformation which provides complete access to the substrate. In the structure of CDK5/p25 complex, S159 which lies within the activation loop, interacts with I153 and E240 in p25, thereby stabilizing an “open” conformation enabling access to the substrate (Tarricone et al., 2001). Tampering with T-loop dynamics would potentially provide a selective means of inhibiting CDK activation, by preventing its conformational reorganization, and thereby substrate binding. We previously tested this hypothesis by developing a conformational biosensor of CDK2 which enabled identification of a novel family of allosteric inhibitors targeting the activation loop (Pellerano et al., 2017). Here we report on the design of a conformational biosensor derived from the protein scaffold of CDK5 into which fluorescent “molecular hinges” were introduced to report on binders or modulators of the activation loop. This biosensor does not respond to ATP or the ATP-competitive inhibitor Roscovitine and was therefore implemented to screen for small molecules that would target CDK5 without binding the ATP pocket and potentially inhibit its activity and glioblastoma cell proliferation, resulting in the discovery of a class of quinazolinone derivatives.

## Materials and Methods

### Design and Engineering of CDKCONF5 Biosensor

The cDNA sequence of human CDK5 was cloned into the pGex6P1 vector (GE Healthcare) 5′BamHI 3′ EcoRI. For CDKCONF5 cysteine residues C53, C83, C94, C117, C290 were mutagenized to serine, so as to yield a mutant bearing three cysteines at positions C157, C190, and C269, within or close to the T-loop of CDK5. CDKCONF5 (C157 C190 C269) : C53S/C83S/C94S/C117S/C290S was expressed in *E.coli* as a GST fusion following induction with 0.5 mM isopropyl β-D-1-thiogalactopyranoside (IPTG) for 5 h at 25°C, then purified by affinity on GST-Trap HP columns (GE Healthcare) followed by size exclusion chromatography on Hiload 16/60 Superdex 75 prepgrade columns (GE Healthcare) equilibrated in PBS buffer (50 mM Phosphate, pH 6.5, 500 mM NaCl), then labeled with a ten-fold molar excess of Cy3 maleimide overnight at 4°C and further purified from free label on NAP-5 columns. CDKCONF2-Cy3 was expressed, labeled and purified as described in Pellerano et al. (2017) and used as a control.

### Protein Expression and Purification of Cyclin-Dependent Kinases

Recombinant GST-CDK5, GST-CIV and 6His-p25 were expressed *in E. coli* following induction with 0.5 mM isopropyl β-D-1-thiogalactopyranoside (IPTG) for 5 h at 25°C (GST-CDK5), or overnight at 20°C (GST-CIV) and purified by chromatography first by affinity GST-Trap HP columns (GE Healthcare) followed by size exclusion chromatography on Hiload 16/60 Superdex 75 prepgrade columns (GE Healthcare) equilibrated in TBS buffer (50 mM TRIS-HCl, pH 7.4, 150 mM NaCl), respectively. Recombinant Tau was expressed in *E. coli* following induction with 0.5 mM IPTG for 3 h at 37°C. The soluble-protein fraction was incubated for 15 min at 75°C and centrifuged to pellet precipitated proteins. The supernatant was incubated with 60% ammonium sulfate overnight. After centrifugation, the pellet was resuspended in TRIS buffer (50 mM TRIS-HCl, pH 7.4, 150 mM NaCl) and purified by FPLC on a HiLoad 16/60 Superdex 75 prep-grade column (GE Healthcare) equilibrated in TRIS buffer.

### Fluorescence Titration Experiments

Fluorescence titration assays were performed in 96-well plates using a Clariostar^TM^ spectrofluorimeter (BMG) in 200 μL PBS (Sigma). Fluorescence emission of Cy3-labeled CDKCONF5 biosensor was acquired at 570 nm following excitation at 544 nm either alone, or following incubation with positive (CIV, Tau) and negative controls (ATP, Roscovitine). Data analysis was performed using the GraFit 7 Software (Erathicus Ltd). Experiments were performed in triplicate, and error bars indicate standard deviation from average.

### Automated High Throughput Screen Conditions and Hit Validation

Prior to the HTS, stability assays and downscaling experiments were performed to establish the best conditions for a miniaturized assay in 96-well plates and optimized so as to obtain a robust and reproducible signal. Performance criteria for reproducibility, sensitivity (in the presence of DMSO), tolerance and robustness were established with the ATP-competitive inhibitor of CDK5 Roscovitine, and a control peptide that barely affected Cy3-labeled CDKCONF5 Biosensor fluorescence and CIV as a positive control, that promotes significant enhancement of Cy3-labeled CDKCONF5 Biosensor fluorescence. Optimal screening conditions were established as follows: 10 nM freshly purified Cy3-labeled CDKCONF5 Biosensor preincubated in 50 mM KH_2_PO_4_/K_2_PO_4_, pH 6.5, 500 mM NaCl, 1% DMSO. Prescreen results: amplication by positive control (CIV) 1.8-fold enhancement; best prescreen Z-factor = 0.89. Prescreen results: Z-factor = 0.54

The screen itself was performed in 96-well black Greiner Bio One plates on a TECAN Freedom Evo Robot with an in-house library of 221 heterocyclic compounds, which belong to several chemical families (quinazolines, diazepines, 1,2,4-triazoles, aminothiazoles.). Fluorescence emission of 10 nM Cy3-labeled CDKCONF5 Biosensor in 50 mM KH_2_PO_4_/K_2_PO_4_, pH 6.5, 500 mM NaCl was measured at 570 nm following excitation at 550 nm after 5 h incubation with 10^−5^ M final concentration each of the 221 compounds. In parallel, the intrinsic fluorescence of the library compounds at 570 nm following excitation at 550 nm was evaluated, to eliminate autofluorescent compounds. Relative increase of fluorescence emission was calculated with reference to the basal fluorescence of Cy3-labeled CDKCONF5 biosensor alone. A molecule was considered a hit when it was not autofluorescent, yet induced changes in Cy3-labeled CDKCONF5 biosensor fluorescence 3 times or greater the standard deviation of CDKCONF5 biosensor fluorescence alone. Hits identified in the screen were retested manually in quadruplet to confirm they were positive hits with a fresh batch of Cy3-labeled CDKCONF5 biosensor and stock solutions of the library compounds in DMSO.

### Synthesis of Quinazolines

**Scheme 1:**
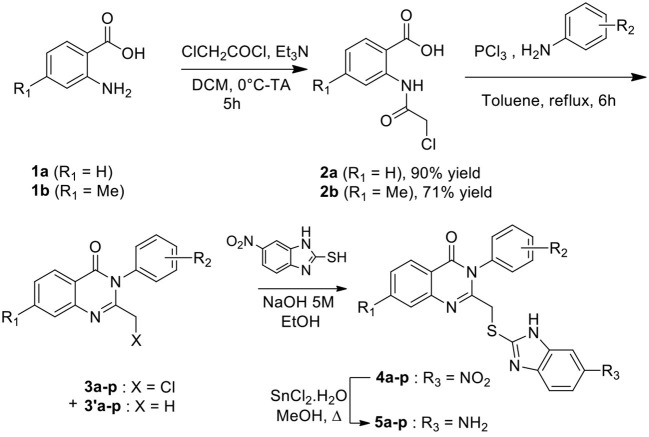
Synthesis of quinazolinone derivatives.

Synthesis of substituted quinazolinones **5a-p** resulted from a 4-steps sequence starting from anthranilic acids (Scheme 1, Table 1, Supplementary Methods). Anthranilic acid **1a** and 4-methylanthranilic **1b** were successively acylated using 2-chloroacetyl chloride to access amides **2a-b** with 90 and 71% isolated yields respectively. Amides **2a-b** reacted then with phosphorus chloride in presence of the suitable aniline, leading to the crude quinazolinone derivatives **3a-p**. A LC-MS analysis of each crude product revealed the concomitant presence of one minor compound, which has been identified as a quinazoline having lost its chlorine atom (compounds **3'a-p**). The crude was then reacted with 2-mercapto-5-nitrobenzimidazole in alkaline medium to offer substituted quinazolinones **4**, which were isolated as a precipitate in a 21–91% isolated yields for the two steps. The reduction of the nitro group was then achieved using SnCl_2_.H_2_O in methanol reflux. After purification by chromatography, amine derivatives **5a-p** were finally isolated in 50–93% yields.

**Table 1:** Compounds synthesized in this study.

**Compounds 3, 4, and 5**	**Correspondence cpd 5/JMV**	**R_**1**_**	**R_**2**_**	**Ratio 3/3'**	**Yield of 4 (%)**	**Yield of 5 (%)**
**a**	JMV5798	H	(3-CF_3_)C_6_H_4_	58/42	54	91
**b**	JMV5891	H	(2-CF_3_)C_6_H_4_	87/13	82	53
**c**	JMV5890	H	(4-CF_3_)C_6_H_4_	92/8	55	53
**d**	JMV5800	H	C_6_H_5_	95/5	83	97
**e**	JMV5888	H	cyclohexyl	56/44	21	75
**3**	JMV5892	H	3-MeC_6_H_4_	94/6	80	54
**g**	JMV5893	H	2-MeOC_6_H_4_	94/6	91	62
**h**	JMV5894	H	3-MeOC_6_H_4_	91/9	65	64
**i**	JMV5883	H	4-MeOC_6_H_4_	92/8	72	91
**j**	JMV5886	H	2-BrC_6_H_4_	87/13	39	50
**k**	JMV5887	H	3-BrC_6_H_4_	80/20	45	77
**l**	JMV5889	H	4-BrC_6_H_4_	51/49	30	59
**m**	JMV5884	H	3-ClC_6_H_4_	90/10	34	74
**n**	–	H	3-NO_2_,4-MeC_6_H_3_	70/30	68	–
**n**	JMV5885	H	3-NH_2_,4-MeC_6_H_3_	–	–	63
**o**	JMV5882	Me	(3-CF_3_)C_6_H_4_	43/57	61	65
**p**	JMV5735	Me	C_6_H_5_	72/28	67	82

### Cell Culture, Cell Proliferation and Cell Extract Preparation

Cell culture media, serum and antibiotics were purchased from Life Technologies. U87 cells were cultured in DMEM + Glutamax supplemented with 10% FCS, 100 units/mL (0.168 mM) penicillin (G sodium salt) and 100 μg/mL (0.172 mM) streptomycin at 37°C in an atmosphere containing 5% CO_2_.

Cell extracts were prepared in PBS lysis buffer containing PBS (Sigma) 0.2 % NP40, 1mM EDTA, 2 mM PMSF, Complete^TM^ protease inhibitors (Roche), and normalized following spectrophometric dosage at 280 nm.

For cell proliferation/viability assays, cells were seeded in 96-well plates at 4,000 cells/well in 100 μL medium. 24 h later, cells were treated in triplicate or quadruplicate with different concentrations of small molecule inhibitors or peptides (from 10 nM to 20 μM). Stock solutions of drugs were prepared in DMSO and freshly diluted in PBS to the desired concentration prior to use, then added to unsynchronized cells cultured to subconfluency (60-70%), which were then further incubated for 24, 48, or 72 h. Cell proliferation was evaluated by crystal violet staining at the indicated times following treatment with drugs: cells were washed with PBS, fixed with 3.7% formaldehyde for 10 min and then incubated with 0.1% crystal violet dye for 30 min. After rinsing, crystals were dissolved in 10% acetic acid and viability was determined by measuring absorbance at 595 nm.

### CDK5 Kinase Activity Assays

CDK5 kinase assays were performed using the CDKACT5 activity biosensor developed in our group and U87 glioblastoma cell extracts as a source of CDK5 kinase (Peyressatre et al., 2020). Fluorescence kinase assays were performed as described previously (Van et al., 2014) in 96-well plates in a thermostated chamber using a Clariostar^TM^ spectrofluorimeter (BMG) at 30°C in 200 μL PBS (Sigma) supplemented with 5 mM MgCl_2_, 0.5 mM ATP. Fluorescence emission of Cy5-labeled CDKACT5 was recorded at 680 nm following excitation at 620 nm. In all experiments, relative fluorescence was calculated following substraction of CDKACT5-Cy5 fluorescence from fluorescence values obtained in the presence of CDK5/p25 kinase incubated with or without inhibitors. Data analysis was performed using the GraFit 7 Software (Erathicus Ltd). Experiments were performed in triplicate, and error bars indicate standard deviation from average.

For concentration-dependent inhibition assays, CDKACT5-Cy5 biosensor was incubated with U87 cell extracts treated with increasing concentrations of each inhibitor and the values corresponding to CDKACT5-Cy5 fluorescence response (at 2000 s) were plotted against inhibitor concentration. When possible, IC_50_ values were calculated using the following equation, which takes into account when maximal inhibition values do not reach zero. In this equation, “Range” is the fitted uninhibited value minus the Background, and s is a slope factor. The equation assumes that y falls with increasing x.

$$\begin{array}{l} y=\frac{Range}{1+\;{\left(\frac{x}{IC_{50}}\right)}^{s}}+Background \end{array}$$

### Statistical Analyses

All data are summarized and presented as the mean ± SEM from at least two or three representative and separate experiments or more whenever indicated. Statistical analyses were performed using a paired Student's *t*-test, with XLSTAT software (Addinsoft, Paris, France). Statistical significance was defined as: ^*^p ≤ 0.05, ^**^p ≤ 0.01 and ^***^p ≤ 0.001.

## Results

### Engineering a Conformational Biosensor to Screen for CDK5 Inhibitors That Do Not Bind the ATP Pocket

**Figure 1:**
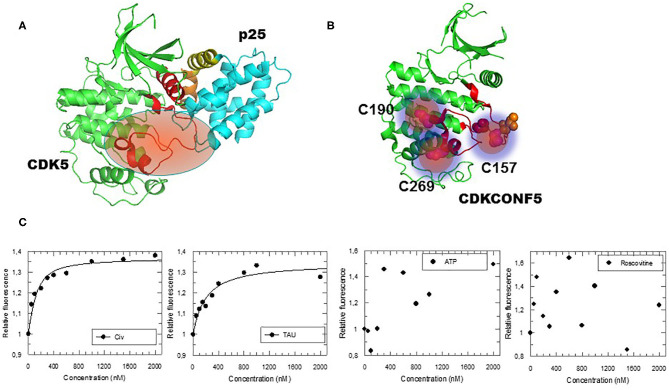
Engineering and Characterization of the CDKCONF5 Biosensor. **(A)** Structure of CDK5/p25 highlighting the activation loop (in red) in an open position enabling complete access to the substrate (PDB 1H4L). **(B)** CDKCONF5 biosensor is derived from the scaffold of CDK5 with 3 cysteine residues C157, C190 and C269 labeled with Cy3 dye (other native cysteines C53, C83, C94, C117, C290 were mutagenized to serine). **(C)** Fluorescence response of CDKCONF5-Cy3 upon titration with CDK-Activating kinase CIV, Tau, ATP or Roscovitine.

In order to screen for compounds that inhibit CDK5 by targeting its activation loop and thereby potentially disrupt its conformational activation, we engineered a fluorescent biosensor according to a previously described strategy (Pellerano et al., 2017) by introducing fluorescent Cy3 dyes into the protein scaffold of CDK5 (cysteine residues C157, C190, and C269, within or close to the T-loop of CDK5), whilst other native cysteines C53, C83, C94, C117, C290 were mutagenized to serine (Figures 1A,B). The CDK5 biosensor (CDKCONF5) responds robustly and in a dose-dependent fashion to the CDK-activating kinase CIV, which phosphorylates the activation loop, and to the CDK5 substrate protein Tau, but is insensitive to ATP and to the ATP-competitive inhibitor Roscovitine (Figure 1C). As such it constitutes a highly sensitive sensor to screen for modulators of CDK5 conformation, whilst discriminating against ATP-competitive compounds.

**Figure 2:**
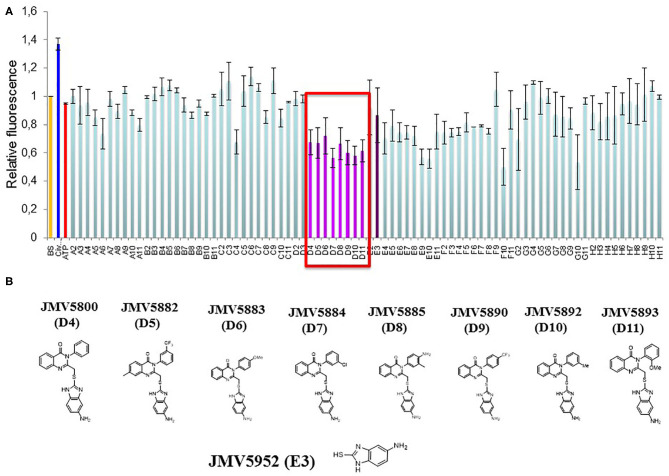
Identification of Quinazoline analogs as hits targeting CDKCONF5. **(A)** Fluorescence response of CDKCONF5 biosensor to compounds on plate 2 of the screen. Fluorescence emission of the biosensor (BS) is in orange; of positive control (CIV) is in dark blue; of negative control (ATP) is in red; and of quinazolinones (compounds in wells D4–D11) in pink/magenta; of 5-aminobenzimidazole (compound in well E3) in purple. **(B)** Structures of quinazolinones (compounds in wells D4–D11) and of 5-aminobenzimidazole (compound in well E3).

In order to apply this tool to screen a library of small molecules, we first performed downscaling experiments to optimize the concentration, temperature and kinetics of the assay and establish the best conditions for a miniaturized assay in 96-well plates to obtain the most robust and reproducible signal and appropriate Z' factor (Supplementary Figures 1, 2). CDKCONF5 biosensor was then applied to screen an in-house library of 221 heterocyclic compounds, from different chemical families including diazepines, quinazolines, 1,2,4-triazoles and aminothiazoles synthesized in our institute (Institut des Biomolecules Max Mousseron, Montpellier, France). The layout of compounds in 96-well plates is shown in Supplementary Figure 2B). As expected, 10 μM ATP used as a negative control did not induce any significant changes in CDKCONF5 fluorescence, whereas 400 nM CIV used as a positive control induced 40% fluorescence enhancement. Unexpectedly, very few compounds induced fluorescence enhancement of CDKCONF5, but several hits induced significant quenching of fluorescence, as shown for the compounds screened in plate 2 (Figure 2A). In particular we identified a family of 8 substituted quinazolinones which induced between 30 and 40% quenching of CDKCONF5 which we selected for further studies. These compounds possess a quinazolinone core, substituted in position 3 by a phenyl group (substituted or not) and bears, in position 2, an aminobenzimidazole group. These compounds are: JMV5800, JMV5882, JMV5883, JMV5884, JMV5885, JMV5890, JMV5892, and JMV5893 (compounds D4–D11 in plate 2). Compound JMV5952, corresponding to the common 5-aminobenzimidazole group present in all selected compounds, did not affect CDKCONF5 fluorescence in the screen (compound E3 in plate 2). This result indicates that this group is not sufficient to modulate conformational transitions of CDKCONF5 biosensor and JMV5952 was therefore considered as a negative control in subsequent experiments. Structures of these compounds are shown in Figure 2B.

Since CDKCONF5 biosensor quenching was unexpected compared to fluorescence enhancement observed with the positive control, we asked whether it might be due to direct interactions with the Cy3 probe since this would lead to quenching. Fluorescence titration of 200 nM Cy3 alone with compound JMV5800 and JMV5884 indeed induced Cy3 quenching inferring that these compounds interacted with CDK5 scaffold at positions where the probe was conjugated (Supplementary Figure 3A). In contrast, these compounds did not induce any significant quenching of the CDKCONF2-Cy3 conformational biosensor, previously developed to screen for CDK2 allosterics (Pellerano et al., 2017) (Supplementary Figure 3B). Since CDKCONF-Cy3 biosensor is derived from CDK2, the most closely related CDK to CDK5, the lack of quenching observed compared to CDKCONF5-Cy3 response supports selective interaction of quinazolines with CDK5 over CDK2. Moreover, these results indicate that the interaction and quenching of the quinazolines with the Cy3 dye is not random but quite specifically defined within the CDKCONF5 scaffold.

In an attempt to address how quinazolines might affect the conformational dynamics of CDKCONF5-Cy3, we asked whether they might compete with p25 binding. To this aim we performed fluorescence titration experiments in which CDKCONF5B-Cy3 was incubated with 400 nM recombinant 6His-p25 and/or a ten-fold greater concentration (4 μM) of quinazolines JMV5800 and JMV5884. Whereas, quinazolines induced significant quenching of CDKCONF5-Cy3 fluorescence, incubation with p25 counteracted this effect, promoting a net relative increase in CDKCONF5-Cy3 fluorescence (Supplementary Figure 4). These data infer that quinazolines may affect the conformational dynamics of CDK5 induced by p25 binding, thereby competing with its site of interaction.

### Quinazolinone Analogs Identified in the CDKCONF5 Screen Modulate CDK5 Activity

**Figure 3:**
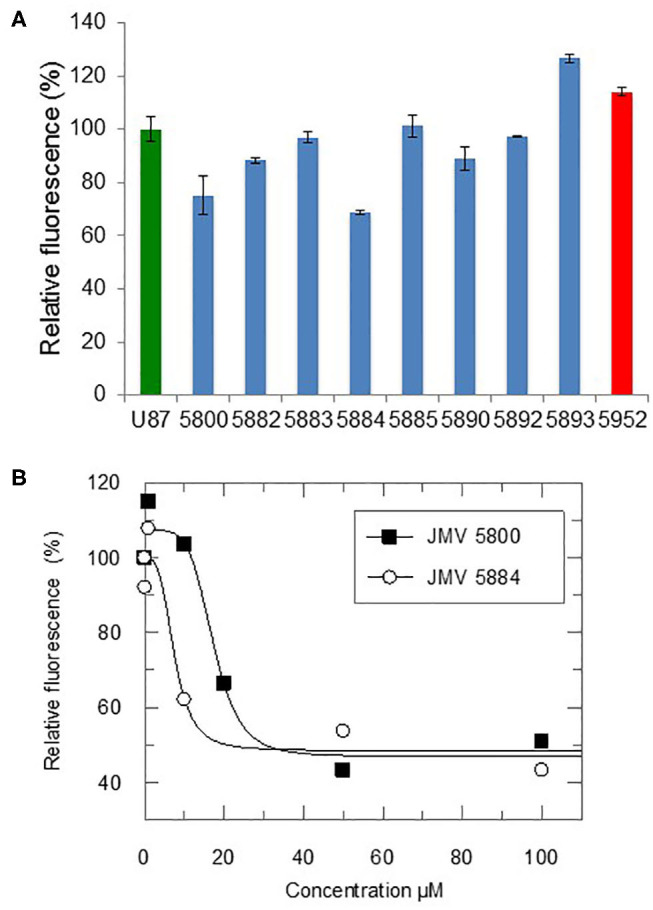
Quinazolinones modulate CDK5 activity. **(A)** Fluorescence-based CDK5 activity assays with the CDKACT5 biosensor, 10 μM quizanolinones and U87 cell extracts. 100% activity for U87 cell extracts alone (green) and relative activity upon incubation with 10uM quizanolinones (blue) or compound 5952 (red). **(B)** Concentration-dependent inhibition of CDK5 activity with JMV5800 and 5884.

In order to determine whether the quinazolinone modulators of CDKCONF5 fluorescence indeed affected CDK5 activity, we performed CDK5 kinase inhibition assays with 10^−5^M of these compounds using the CDKACT5 activity biosensor developed in our group and U87 glioblastoma cell extracts as a source of CDK5 kinase (Peyressatre et al., 2020) (Figure 3A). In this assay, upon incubation with a source of CDK5 kinase, in this case U87 cell extracts, fluorescence of the CDKACT5 biosensor is enhanced, thereby reporting on CDK5 activity (Supplementary Figure 5A). However, when incubated with 10 μM quinazolinones identified in the screen, we found that only two of these compounds effectively inhibited CDK5 activity: JMV5800 and JMV5884 (Figure 3A, Supplementary Figure 5B). Interestingly, compounds JMV5952 and JMV5893 promoted further increase in biosensor fluorescence inferring a possible potentiation of CDK5 activity. To further characterize the efficiency of JMV5800 and JMV5884, we performed a concentration-dependent inhibition assay (Figure 3B), which enabled calculation of IC_50_ values of 8.29 ± 3.56 μM and 7.6 ± 3.8 μM, respectively and a maximal inhibitory efficiency of 50-60% at 100 μM (Table 2).

**Table 2:** Relative CDK5 activity in lysates of U87 cells treated with 100 μM inhibitors.

**Molecules**	**Activity at 100 μM (%)**
*JMV 5800*	50.7 ± 7.8
*JMV5884*	43.2 ± 5.6
*JMV5735*	33.5 ± 1
*JMV5798*	40.2 ± 2.4
*JMV5886*	45 ± 0.6
*JMV5887*	76.5 ± 6.4
*JMV 5888*	80 ± 5.5
*JMV5889*	53.6 ± 11.2
*JMV 5891*	75.5 ± 1.3
*JMV 5894*	68.3 ± 2.6

### Characterization of Quinazolinone Derivatives as Inhibitors of CDK5 Activity

**Figure 4:**
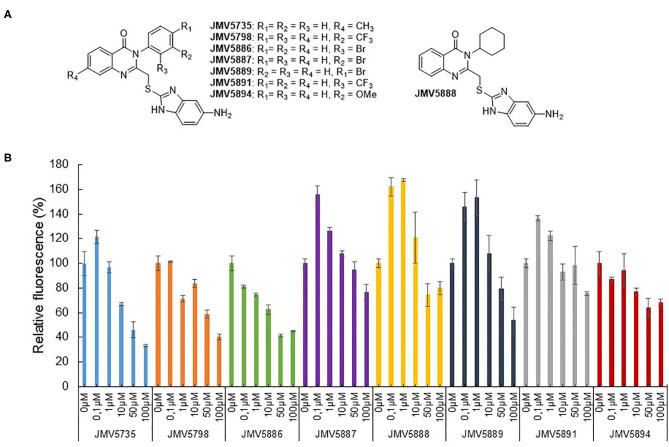
Quinazolinone derivatives inhibit CDK5 activity. **(A)** Quinazolinone derivatives synthesized. **(B)** Concentration-dependent inhibition of CDK5 activity measured with the CDKACT5 biosensor and U87 cell extracts (JMV5735, JMV5798, JMV5886, JMV5887, JMV5888, JMV5889, JMV5891, and JMV5894).

Based on the results obtained with the hits identified in the screen, a second series of quinazolinone derivatives were synthesized and evaluated. Four kinds of modulations were studied: first, importance of the methyl group in position 7 of the quinazoline ring, second, replacement of the aromatic group in N-3 by a cyclohexyl group, and third, the influence of the nature and of the position of the substituent on the phenyl group (Figure 4A). Fluorescence activity assays were performed with the CDK5 activity reporter and U87 cell extracts, to assess the inhibitory potential of the quinazoline derivatives between 100 μM and 100 nM (Figure 4B). The eight quinazoline derivatives, bearing the aminobenzimidazole moiety, displayed inhibitory potential toward CDK5 activity in U87 cell extracts (JMV5735, JMV5798, JMV5886, JMV5887, JMV5888, JMV5891, JMV5894, and JMV5889). Table 2 lists the maximal inhibition values of CDK5 activity by these compounds at 100 μM using U87 cell extracts as a source of CDK5 activity. Compounds JMV5735, 5798, 5886 and 5889 achieved 66.5, 60, 55, and 46% inhibition at 100 μM, respectively. IC_50_ values of 9.35 ± 11.1, 24.4 ± 32.5, 28.7 ± 51.2 μM were calculated for JMV5735, 5886, and 5889, respectively (Supplementary Figure 6). Hence, the most promising compounds with respect to *in vitro* kinase inhibition were JMV5735, 5886, 5889. JMV 5798 values were unfittable to calculate an IC_50_ value. Compounds JMV5887, 5888, 5891, and 5894 inhibited kinase activity less significantly (20–33% maximal inhibition at 100 μM concentration quinazolinones) (Figure 4B, Table 2).

### Characterization of Quinazoline Derivatives as Inhibitors of U87 Glioblastoma Cell Proliferation

**Figure 5:**
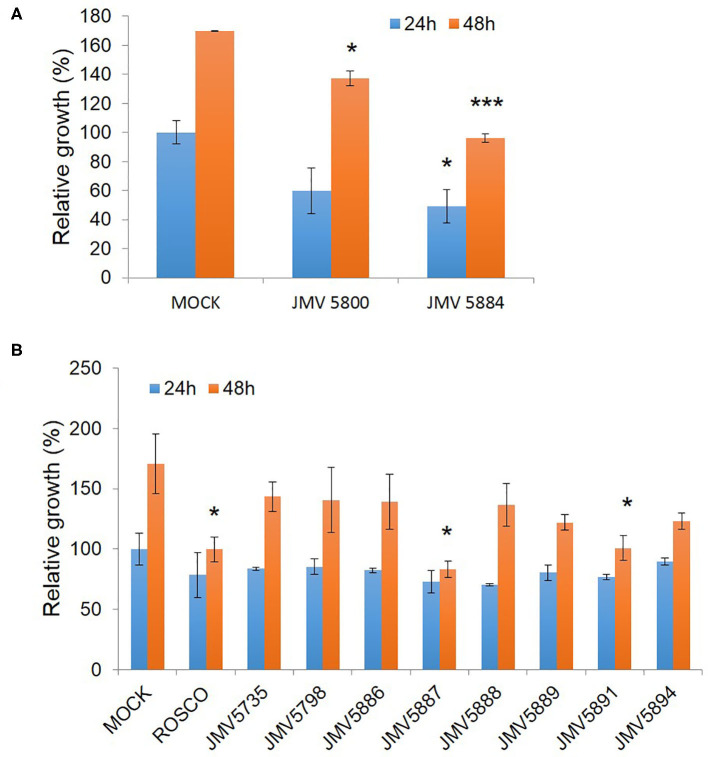
Quinazolinone derivatives inhibit proliferation of U87 glioblastoma cells. **(A)** Proliferation Assays with 10 μM hits JMV5800 and JMV5884. **(B)** Proliferation assays with quinazolinone derivatives 10 μM JMV5735, JMV5798, JMV5886, JMV5887, JMV5888, JMV5891, JMV5894, and JMV5889. Statistical significance: *p ≤ 0.05, **p ≤ 0.01, and ***p ≤ 0.001.

We finally addressed the inhibitory potential of these quinazolinones compared to Roscovitine in proliferation assays in the U87 glioblastoma cell line (Figure 5). The original hits identified in the screen, JMV5800 and JMV5884 were efficient inhibitors of U87 cell proliferation with 40% and 50% inhibition, respectively, but JMV5800 was less potent than JMV5884 after 48 h. Likewise, the eight other quinazolinones derivatives characterized *in vitro*, induced a similar inhibition of U87 cell proliferation as compared to JMV5800 and JMV5884 and to Roscovitine at 24 h, relative to mock-treated cells. After 48 h however further differences were observed with compounds JMV5887 and JMV5891 exhibiting the greatest anti-proliferative potential, comparable to that of JMV5884 and Roscovitine. JMV5889 and 5894 inhibited cell proliferation less potently than Roscovitine after 48h, but still significantly relative to mock-treated cells followed by JMV5888, 5886, 5798, and 5735 which induced similar inhibition as JMV5800. U87 cell proliferation values relative to mock-treated cells following treatment with quinazolinone derivatives after 24 and 48 h are listed in Table 3.

**Table 3:** Proliferation assays: ^*a*^Mock in the first set of proliferation assays performed with JMV5800 and JMV5884; ^*b*^Mock in the second set of proliferation assays performed with quinazolinone derivatives.

**Molecules**	**24 h Proliferation (%)**	**48 h Proliferation (%)**
*MOCK^*a*^*	100 ± 8.03	169.83 ± 0.35
*JMV 5800*	59.9 ± 15.7	137.28 ± 5.17
*JMV5884*	49.19 ± 11.5	96.1 ± 3.07
*MOCK^*b*^*	100 ± 13.1	170.53 ± 24.9
*Roscovitine*	78.38 ± 18.8	99.66 ± 10.1
*JMV5735*	83.8 ± 1.3	143.35 ± 12
*JMV 5798*	85.33 ± 6.4	138.23 ± 26.8
*JMV5886*	82.26 ± 2	139.05 ± 23
*JMV5887*	73.06 ± 9.3	83.39 ± 6.8
*JMV5888*	70.5 ± 1	136.29 ± 17.7
*JMV5889*	80.32 ± 6.2	121.86 ± 4.1
*JMV5891*	76.64 ± 2.2	100.68 ± 10.4
*JMV5894*	89.84 ± 2.9	122.99 ± 6.9

## Discussion

CDK5/p25 kinase plays a major role in neuronal functions, and is hyperactivated in several human cancers including glioblastoma and neurodegenerative pathologies such as Alzheimer's and Parkinson's. Several inhibitors of CDK5 have been reported in the literature, including pan-ATP-competitive inhibitors such as Roscovitine, or more selective inhibitors such as Dinaciclib. To date all compounds targeting CDK5 which have made it through *in vivo* and preclinical studies bind the ATP binding site, which exhibits similarity throughout the kinome and is further found in a many other enzymes including ATP-binding chaperones and molecular motors. As a consequence, these inhibitors display limited selectivity and face important limitations, since their promiscuity affords for severe and undesirable toxic side-effects and poor tolerability, the dose administered necessary to inhibit the kinase target often inducing off-target effects that limit efficacy (Guha, 2013). Aside from ATP-competitive compounds alternative strategies have been explored to interfere with pockets and interfaces other than the ATP binding pocket of the CDK, including peptides and small molecules, some of which exhibit potent antiproliferative activity, although none of them has yet made it to the clinic.

To address this challenge and propose new strategies to target CDK5, we engineered a CDK5-specific conformational biosensor designed to discriminate against ATP-pocket binders and to report on modulators of the activation segment. By implementing this biosensor to screen for compounds that target conformational activation of CDK5, we identified a family of quinazolinone derivatives which induced biosensor quenching rather than enhancement as expected by comparison with the positive control (CIV). Fluorescence titration of Cy3 with these compounds revealed that they induce probe quenching and therefore directly interact with Cy3, inferring that they bind close to the three cysteines onto which they are conjugated within CDKCONF5 scaffold (Cys 157, C190, C269). In contrast these compounds do not affect fluorescence of the CDKCONF2 conformational biosensor we previously developed, inferring that these compounds bind CDK5 selectively over the closely related CDK2. In addition preliminary mechanistic studies suggest that these compounds most likely compete with the site of interaction of p25 with CDK5. We further showed that several of these compounds indeed inhibited CDK5 kinase activity *in vitro* using U87 cell extracts and further exhibited inhibitory potential toward glioblastoma cell proliferation with an efficacy in the same range as Roscovitine.

All hits identified in the screen and derivatives synthesized thereafter were substituted by a 5-aminobenzimidazole group in position 2 of the quinazoline and several kind of substitutions at N3-phenyl group were tolerated. The common 5-aminobenzimidazole-2-thiol alone revealed to be inactive (JMV5952). Moreover, introduction of a methyl group at R4 (Figure 4A) led to a decrease of the kinase inhibition (JMV5735 was less active than JMV5800). Replacement of the N3-phenyl group by a cyclohexyl moiety abolished activity completely, indicating that an aromatic group was necessary at position 3 of the quinazoline ring. Considering the nature of the substituent carried out by the phenyl group, a chlorine (JMV5884), a bromine (JMV5889 and JMV5886) or a methoxy group (JMV5894) were tolerated but not a trifluoromethyl substituent (for example JMV5891 inhibited CDK5 activity but led to a low inhibition of U87 cell proliferation). Halogen atoms possess both -I electron withdrawing and +M mesomer donor effects, whereas methoxy is +M mesomer donor group and trifluoromethyl group is a -I electron withdrawing group. Our results suggests that the activity was dependent on lack of substitution of the N-3 phenyl group (JMV5800) or substitution by a mesomer donor group. Finally, the position of the substituent did not appear to be crucial for activity.

Although compounds JMV5887 and 5891 were the most potent inhibitors of cell proliferation, their *in vitro* efficacy toward CDK5 activity was not as significant as quinazolinones JMV5800, JMV5884, JMV5735, JMV5886, and JMV5889. Conversely, except for JMV5884, these compounds did not achieve the most significant inhibition of U87 cell proliferation. Considering that the *in vitro* kinase inhibition assays point to specific activity of compounds on the target of interest, and taking together the maximal relative inhibition potential of compounds in these kinase assays and in cell proliferation assays, JMV5884 appears to offer the most promising potential combining both significant inhibition of both CDK5 activity and U87 cell proliferation, followed by JMV5889 then JMV5735, JMV5798 JMV5886, and JMV5894. JMV5884 has a chlorine substitution on the N-3 phenyl relative to JMV5800 (unsubstituted phenyl group). Likewise JMV5889 and JMV5886 have a bromide atom on this ring (*para* and *ortho* position respectively) and JMV5894 has a meta methoxy group on the N-3 phenyl group.

Several quinazolines have been evaluated as anticancer therapeutics in different cancer cell lines and EGFR has been identified as one of the relevant molecular targets (Khodair et al., 2019; Soliman et al., 2019). Sulphonamide benzoquinazolinones have also been reported as dual EGFR/HER2 inhibitors with micromolar IC_50_ values, apoptosis inducers and radiosensitizers. Quinazalinone derivatives have also been identified as Pan-Pim kinase inhibitors (Pettus et al., 2016), PI3K inhibitors (Ma et al., 2018), ALK2 inhibitors (Hudson et al., 2018). More recently a quinazolinone-based dual inhibitor of CDK4 and tubulin polymerization has been reported for anti-cancer therapy (Sonawane et al., 2019). Hence there is a strong potential for quinazolines to bind protein kinases, in line with our findings that several quinazolinone derivatives constitute CDK5 inhibitors. The conformational biosensor-based screening approach we have developed has yielded the first small molecules reported to target CDK5 at a site other than the ATP pocket. These compounds constitute templates for development of more potent compounds, which can be developed through standard structure/activity relationship studies to identify derivatives with improved inhibitory potential. Our approach constitutes a very potent means of identify non-ATP competitive kinase inhibitors, as we also reported in a previous study yielding original inhibitors of CDK2 (Pellerano et al., 2017), since it discriminates against ATP-pocket binders and is based on the design of a biosensor that reports on conformational transitions required for kinase activation. The consistency of this approach relies on an in-depth understanding of the molecular mechanism of activation of the kinase of interest as well as structural information.

## Data Availability Statement

All datasets generated for this study are included in the article/Supplementary Material.

## Author Contributions

MPey, JG-V, and MPel generated, purified and characterized the CDKCONF5 biosensor *in vitro* in fluorescence titration experiments, established the conditions for the HTS and performed the screen. MPey, AL, and MPel performed fluorescence validation experiments. MPey, JG-V, and MM analyzed the results from the screen and determined hit compounds to be characterized further. MPey performed kinase assays and cell proliferation assays. NM and VL provided compounds of the in-house library. NM and DA synthesized hit derivatives. MM designed CDKCONF5 biosensors and its implementation for HTS and coordinated the study together with VL and NM. MM, AL, MPel, and NM wrote the manuscript. All authors contributed to the article and approved the submitted version.

## Conflict of Interest

The authors declare that the research was conducted in the absence of any commercial or financial relationships that could be construed as a potential conflict of interest.
