# Supplementary material for: Identification of Quinazolinone Analogs Targeting CDK5 Kinase Activity and Glioblastoma Cell Proliferation
Source: Front Chem. 2020 Aug 19;8:691. doi: 10.3389/fchem.2020.00691 (PMC7466635; doi:10.3389/fchem.2020.00691)
Supplement: Supplementary file 1 [file Table_1.docx]

Supplementary Material

# Supplementary Figures

**Supplementary Figure 1 -** Fluorescence response of CDKCONF5-Cy3 biosensor to CIV (200 nM and 400 nM) and ATP (1 μM and 10 μM) in PBS / 1% DMSO) at 30 ºC in a Clariostar^TM^ fluorimeter. A : 50 nM CDKCONF5-Cy3; B. 25 nM CDKCONF5-Cy3; C. 10 nM CDKCONF5-Cy3

**Supplementary Figure 2 – A.** Fluorescence response of 10 nM CDKCONF5B-Cy3 to CIV (200 nM and 400 nM) and ATP (10 μM) in PBS / 1% DMSO) at 20 and 30 ºC in a Tecan^TM^ fluorimeter used for the screen B. Layout of biosensor, positive and negative controls in the plates for the screen.

**Supplementary Figure 3 –** A. Fluorescence titration of 200 nM Cy3 with increasing concentrations of JMV5800 and JMV5884. B. Fluorescence titration of CDKCONF2-Cy3 with the 10 μM quinazolines identified in the primary screen with CDKCONF5-Cy3 and in the same conditions, following 5h incubation at 25°C.

**A.**

**B.**

**Supplementary Figure 4 –**Fluorescence titration of CDKCONF5-Cy3 with p25 and JMV5800 or JMV5884.

**Supplementary Figure 5 –** A. CDK5 activity in U87 cell extracts – fluorescence based assay with the CDKACT5-Cy5 activity biosensor B. Evaluation of 10 μM quinazolinones on CDK5 activity in U87 cell extracts

**Supplementary Figure 6 -** A. Dose-dependent inhibition curves of CDK5 activity in U87 cell extracts for determination of IC50

# Supplementary methods

Commercially available reagents and solvents were used without further purification. Reactions were monitored by HPLC using an analytical Chromolith Speed Rod RP-C18 185 Pm column (50x4.6 mm, 5 mm), at a flow rate of 3.0 mL/min, and gradients of 100/0 to 0/100 eluents A/B for 5 min (eluent A = H_2_O/0.1% TFA and B = CH_3_CN/0.1% TFA). Detection was performed at 214 nm using a photodiode array detector. Retention times (Tr) are reported in min. ^1^H and ^13^C NMR spectra were recorded at room temperature in deuterated solvents. Chemical shifts (δ) are expressed in parts per million (ppm), relative to the resonance of CDCl_3_ = 7.26 ppm for ^1^H (77.16 ppm for ^13^C), CD_3_OD = 3.31 ppm for ^1^H (49.00 ppm for ^13^C) or DMSO *d*_6_ = 2.50 ppm for ^1^H (39.52 ppm for ^13^C). The following abbreviations were used: s (singlet), d (doublet), t (triplet), q (quartet), m (multiplet), br (broad). Analytical thin-layer chromatography (TLC) was performed using 60 F254 aluminium-backed silica gel plates coated with a 0.2 mm thickness of neutral aluminium oxide. LC-MS spectra (ESI) were recorded using an analytical Chromolith Speed Rod RP-C18 185 Pm column (50 x 4.6 mm, 5 mm); solvent A, H_2_O/HCOOH 0.1%; solvent B, CH_3_CN/HCOOH 0.1%; gradient, 0% solvent B to 100% solvent B in solvent A in 3 min; flow rate, 3.0 mL/min. High-resolution mass spectrometry analyses were performed with a time-of-flight mass spectrometer fitted with an electrospray ionisation source. All measurements were performed in the positive ion mode. Melting points (Mp) are uncorrected and were recorded on a capillary melting point apparatus.

**Synthesis of compounds 2 :**

To a solution of 2 g of anthranilic acid **1a** or **1b** in 20 mL of dichloromethane was added one equivalent of triethylamine. The reaction was stirred and cooled at 0°C. One equivalent of chloroacetyl chloride was then added dropwise during 15 min. The mixture was then stirred at room temperature (r.t.) for 6 h. After completion of the reaction, the precipitate was filtered and washed with water and then dried under vacuum to offer compounds **2** as white powders.

2-(2-chloroacetamido)benzoic acid **2a**: white powder (2.68 g; 90% yield); mp: 183°C (litt.^[1]^:183-185°C); ^1^H NMR (CDCl_3_, 300 MHz): δ ppm 4.22 (s, 2H), 7.18 (t, 1H, *J* = 9.0 Hz), 7.60 (t, 1H, *J* = 9.0 Hz), 8.15 (dd, 1H, *J* = 9.0, 3.0 Hz), 8,71 (d, 1H, *J* = 9.0 Hz), 11,95 (bs, 1H) ; ^13^C NMR (CDCl_3_, 75 MHz): δ ppm 43,4, 120,6, 123,8, 127,8, 132,0, 135,2, 140,9, 164,3, 165,4; HPLC, Tr = 2,20 min ; MS (ESI^+^): calc. for C_9_H_9_NO_3_Cl: 214.0, found 214.1 [M+H]^+^.

2-(2-chloroacetamido)-4-methylbenzoic acid **2b**: white powder (2.08 g, 71% yield); mp: 208°C (litt.^[2]^: 208-209°C); ^1^H NMR (CDCl_3_, 300 MHz): δ ppm 2.35 (s, 3H), 4.42 (s, 2H), 7.00 (d, 1H, *J* = 8.1 Hz), 7.88 (d, 1H, *J* = 8.1 Hz), 8.31 (s, 1H); ^13^C NMR (CDCl_3_, 75 MHz): δ ppm 22.4, 43.4, 112.4, 121.1, 124.8, 132.0, 140.7, 147.2, 165.3 171.4; HPLC, Tr = 2.75 min; MS (ESI^+^): calc. for C_10_H_11_NO_3_Cl: 228.0, found 228.2 [M+H]^+^.

**Synthesis of quinazolines 3** and **3'**

To a solution of 1.4 mmol of compound **2a** or **2b** in 10 mL of toluene, were added 7 mmol of PCl_3_ (5 equiv.) and 2.1 mmol of the suitable aniline (1.5 equiv.). The mixture was stirred at reflux for 6-10 h (monitoring by HPLC). After completion of the reaction and cooling to r.t., the mixture was evaporated to dryness and 10 mL of water were added. The reaction mixture was neutralized with the addition of sodium bicarbonate and extracted with chloroform (4 x 25 mL). The organic layers were combined, dried over Na_2_SO_4_, filtered and evaporated under vacuum to offer a mixture of **3** + **3'** which was used without furher purification in the next step.

**Synthesis of substituted quinazolines 4 :**

To a solution of 1 mmol of quinazoline **3**+**3'** in 10 mL of ethanol, were added 1 mmol of 2-mercapto-5-nitrobenzimidazole (195 mg, 1 equiv.) and 0.2 mL of a 5N NaOH aqueous solution. The mixture was stirred at r.t. for 2 h. After reaction completion, the precipitate was filtered and washed with diethylic ether to offer compounds **4**.

2-(((5-nitro-1H-benzo[*d*]imidazol-2-yl)thio)methyl)-3-(3-(trifluoromethyl)-phenyl)quinazolin-4(3H)-one **4a**: yellow solid (0.159 g, 32% yield); ^1^H NMR (CDCl_3_, 400 MHz) : δ ppm 4.45 (dd, 2H, *J* = 20.0, 16.0 Hz), 7.53 (d, 1H, *J* = 8.0 Hz), 7.57 (t, 1H, *J* = 8.0 Hz ), 7.71 (d, 1H, *J* = 8.0 Hz), 7.76-7.83 (m, 2H), 7.89 (t, 1H, *J* = 8.0 Hz), 7,95 (d, 1H, *J* = 8.0 Hz), 8.02 (d, 1H, *J* = 8.0 Hz), 8.10 (s, 1H), 8.14 (d, 1H, *J* = 8.0 Hz), 8.23 (s, 1H), 13.28 (s, 1H); ^13^C NMR (CDCl_3_, 100 MHz): δ ppm 35.9, 106.7, 110.6, 113.0, 117.6, 120.6, 122.3, 125.3, 126.3, 126.5, 127.0, 127.4, 130.0, 130.4, 130.8, 133.5, 135.0, 137.2, 140.2, 146.6, 151.9, 154.4, 161.2; HPLC, Tr = 3.07 min; MS (ESI^+^): calc. for C_23_H_15_F_3_N_5_O_3_S: 498.1, found 498.2 [M+H]^+^.

2-(((5-nitro-1H-benzo[*d*]imidazol-2-yl)thio)methyl)-3-(2-(trifluoromethyl)-phenyl)quinazolin-4(3H)-one **4b**: yellow solid (0.407g; 82% yield); ^1^H NMR (DMSO-*d*_6_, 400 MHz) : δ ppm 4.44 (ABX, 2H), 7.55 (d, 1H, *J* = 8.0 Hz), 7.61 (t, 1H, *J* = 8.0 Hz), 7.72 (d, 1H, J = 8.0 Hz), 7.76 (t, 1H, J = 8.0 Hz), 7.80-7.93 (m, 3H), 8.01-8.06 (m, 2H), 8.15 (dd, 1H, J = 8.0, 4.0 Hz), 8,24 (d, 1H, J = 4.0 Hz), 13,30 (bs, 1H) ; ^13^C NMR (DMSO-*d*_6_, 100 MHz) : δ ppm 35.6, 117.5, 119.0, 120.2, 121.7, 124.5, 126.0, 126.3, 126.6, 126.9, 127.1, 127.8, 127.9, 130.8, 131.9, 133.8, 134.5, 135.4, 142.2, 144.6, 146.4, 151.7, 161.0; HPLC, Tr = 3.35 min; MS (ESI+): calc for C_23_H_15_F_3_N_5_O_3_S: 498.1, found 497.9 [M+H]^+^.

2-(((5-nitro-1H-benzo[*d*]imidazol-2-yl)thio)methyl)-3-(4-(trifluoromethyl)-phenyl)quinazolin-4(3H)-one **4c**: yellow solid (0.273 mg; 55% yield); ^1^H NMR (DMSO-*d*_6_, 400 MHz): δ ppm 4.45 (s, 2H), 7.53 (d, 1H, *J* = 12.0 Hz), 7,58 (t, 1H, *J* = 8.0 Hz), 7,72 (d, 1H, *J* = 8.0 Hz), 7.85-7.93 (m, 6H), 8.02 (dd, 1H, *J* = 8.0, 4.0 Hz), 8,13 (d, 1H, *J* = 4.0 Hz), 8.23 (s, 1H) ; ^13^C NMR (DMSO-*d*_6_, 100 MHz) : δ ppm 36.0, 117.4, 120.6, 125.2, 126.5, 126.7 (2C), 127.0, 127.4, 127.9, 129.2, 129.6, 129.9, 130.3 (2C), 133.5, 135.1, 140.2, 142.1, 146.6, 151.7, 155.3, 161.1; HPLC, Tr = 3,38 min; MS (ESI^+^): calc for C_23_H_15_F_3_N_5_O_3_S: 498.1, found 498.0 [M+H]^+^.

2-(((5-nitro-1H-benzo[*d*]imidazol-2-yl)thio)methyl)-3-phenylquinazolin-4(3H)-one **4d**: yellow solid (0.356 g, 83% yield); ^1^H NMR (DMSO-*d*_6_, 400 MHz) : δ ppm 4.4 (s, 2H), 7.50-7.59 (m, 7H), 7.69 (d, 1H, J = 8.0 Hz), 7,85 (t, 1H, J = 12.0 Hz), 8,03 (d, 1H, J = 12.0 Hz), 8.13 (d, 1H, J = 12.0 Hz), 8,25 (s, 1H), 13,25 (s, 1H) ; ^13^C NMR (DMSO-*d*_6_, 100 MHz) : δ ppm 36.1, 110.3, 113.4, 117.2, 120.7, 126.5, 126.9, 127.2, 128.8 (2C), 129.4, 129.6 (2C), 134.8, 136.5, 139.6, 141.9, 144.0, 146.6, 152.1, 155.9, 161.1; HPLC, Tr = 3,07 min; MS (ESI^+^): calc for C_22_H_17_N_5_O_3_S 430.1, found 430.1 [M+H]^+^.

2-(((5-nitro-1H-benzo[*d*]imidazol-2-yl)thio)methyl)-3-(*m*-tolyl)quinazolin-4(3H)-one **4f**: beige solid (0.354 g, 80% yield); ^1^H NMR (DMSO-*d*_6_, 300 MHz) : δ ppm 2.34 (s, 3H), 4.44 (ABX, 2H), 7.28 (d, 1H, *J* = 9.0 Hz), 7.36-7.48 (m, 3H), 7.57 (t, 2H, *J* = 9.0 Hz), 7,69 (d, 1H, *J* = 9.0 Hz), 7,86 (td, 1H, *J* = 9.0, 3.0 Hz), 8,02 (dd, 1H, *J* = 9.0, 3.0 Hz), 8,12 (dd, 1H, *J* = 9.0, 3.0 Hz), 8,24 (s, 1H) ; ^13^C NMR (DMSO-*d*_6_, 75 MHz) : δ ppm 20.8, 36.0, 98.4, 117.4, 120.6, 125.8, 126.4, 126.9, 127.2, 129.2, 129.5, 130.0, 130.5, 131.9, 134.8, 135.5, 136.1, 136.3, 139.1, 142.1, 146.6, 152.2, 161.0; HPLC, Tr = 3.19 min; MS (ESI+): calc. for C_23_H_19_N_5_O_3_S 444.1, found 444.0 [M+H]^+^.

3-(2-methoxyphenyl)-2-(((5-nitro-1H-benzo[*d*]imidazol-2-yl)thio)methyl)-quinazolin-4(3H)-one **4g**: beige solid (0.418 g, 91% yield); ^1^H NMR (DMSO-*d*_6_, 400 MHz): δ ppm 3.78 (s, 3H), 4.44 (ABX, 2H), 7,12 (td, 1H, *J* = 8.0, 4.0 Hz), 7,26 (dd, 1H, *J* = 8.0, 4.0 Hz), 7.47-7.60 (m, 4H), 7,70 (d, 1H, *J* = 4.0 Hz), 7,88 (td, 1H, *J* = 8.0, 4.0 Hz), 8,03 (dd, 1H, *J* = 8.0, 4.0 Hz), 8,12 (dd, 1H, *J* = 8.0, 4.0 Hz), 8,25 (d, 1H, *J* = 4.0 Hz), 13,66 (bs, 1H) ; ^13^C NMR (DMSO-*d*_6_, 100 MHz): δ ppm 35.5, 55.9, 112.7, 113.0, 117.5, 120.5, 121.0, 124.4, 126.5, 127.0, 127.4, 129.0, 129.4, 130.1, 131.3, 132.8, 135.0, 142.1, 146.6, 147.0, 152.7, 154.4, 160.6; HPLC, Tr = 3,08 min; MS (ESI+): calc. for C_23_H_19_N_5_O_4_S 460.1, found 460.0 [M+H]^+^.

3-(3-methoxyphenyl)-2-(((5-nitro-1H-benzo[*d*]imidazol-2-yl)thio)methyl)-quinazolin-4(3H)-one **4h**: beige solid (0.298 g, 65% yield); ^1^H NMR (DMSO-*d*_6_, 400 MHz) : δ ppm 3.77 (s, 3H), 4.47 (s, 2H), 7.05 (ddd, 1H, *J* = 8.0, 4.0, 0.8 Hz), 7.14 (ddd, 1H, *J* = 8.0, 4.0, 0.8 Hz), 7.20 (t, 1H, *J* = 4.0 Hz), 7.48 (t, 1H, *J* = 8.0 Hz), 7.57 (td, 2H, *J* = 8.0, 4.0 Hz), 7.69 (d, 1H, *J* = 8.0 Hz), 7.86 (td, 1H, *J* = 8.0, 4.0 Hz), 8,03 (dd, 1H, *J* = 8.0, 4.0 Hz), 8.13 (dd, 1H, *J* = 8.0, 4.0 Hz), 8.25 (d, 1H, *J* = 4.0 Hz), 13.66 (bs, 1H) ; ^13^C NMR (DMSO-*d*_6_, 100 MHz) : δ ppm 36.0, 55.4, 114.6, 115.1, 117.5, 119.4, 120.7, 120.9, 126.5, 126.9, 127.3, 129.4, 130.4, 134.9, 137.5, 140.9, 142.1, 143.9, 146.6, 152.2, 155.6, 160.1, 161.0; HPLC, Tr = 3.04 min; MS (ESI+): MS (ESI+): calc. for C_23_H_19_N_5_O_4_S 460.1, found 460.0 [M+H]^+^.

3-(4-methoxyphenyl)-2-(((5-nitro-1H-benzo[*d*]imidazol-2-yl)thio)methyl)-quinazolin-4(3H)-one **4i**: beige solid (0.330 g, 72% yield); ^1^H NMR (DMSO-*d*_6_, 400 MHz) : δ ppm 3.76 (s, 3H), 4.45 (s, 2H), 7,05 (dd, 2H, *J* = 8.0, 4.0 Hz), 7,45-7,49 (m, 2H), 7,56 (td, 2H, *J* = 8.0, 4.0 Hz), 7,69 (d, 1H, *J* = 8.0 Hz), 7,86 (td, 1H, *J* = 8.0, 4.0 Hz), 8,03 (d, 1H, *J* = 8.0, 4.0 Hz), 8,12 (dd, 1H, *J* = 8.0, 4.0 Hz), 8,26 (bs, 1H), 13,64 (bs, 1H) ; ^13^C NMR (DMSO-*d*_6_, 100 MHz) : δ ppm 36.2, 55.3, 110.7, 112.9, 114.7 (2C), 117.5, 119.7, 120.7, 120.9, 126.5, 126.9, 127.2, 128.8, 130.0 (2C), 134.8, 140.3, 142.2, 146.6, 152.7, 159.6, 161.4; HPLC, Tr = 3,03 min; MS (ESI+): calc. for C_23_H_19_N_5_O_4_S 460.1, found 460.0 [M+H]^+^.

3-(2-bromophenyl)-2-(((5-nitro-1H-benzo[*d*]imidazol-2-yl)thio)methyl)-quinazolin-4(3H)-one **3j**: yellow solid (0.198 g, 39% yield); ^1^H NMR (DMSO-*d*_6_, 400 MHz): δ ppm 4.44 (ABX, 2H), 7.46 (td, 1H, *J* = 9.0, 3.0 Hz), 7,55 (d, 1H, *J* = 9.0 Hz), 7.58-7.64 (m, 2H), 7.73 (d, 1H, *J* = 9,0 Hz), 7.82 (dd, 1H, *J* = 6.0, 3.0 Hz), 7.87-7.95 (m, 2H), 8.03 (dd, 1H, *J* = 9.0, 3.0 Hz), 8,17 (dd, 1H, *J* = 9.0, 3.0 Hz), 8,25 (d, 1H, *J* = 3.0 Hz), 13,25 (s, 1H); ^13^C NMR (DMSO-*d*_6_, 100 MHz) : δ ppm 36.1, 97.3, 117.5, 120.4, 122.5, 126.6, 127.1, 127.7, 129.0, 129.3, 131.2, 131.7, 133.5, 135.3, 135.4, 141.6, 142.2, 146.5, 151.5, 155.0, 160.3, 162.3; HPLC, Tr = 3,31 min; MS (ESI+): calc. for C_22_H_15_N_5_O_3_S 508.0, found 508.0 [M+H]^+^, 510.0 [M+2+H]^+^.

3-(3-bromophenyl)-2-(((5-nitro-1H-benzo[*d*]imidazol-2-yl)thio)methyl)-quinazolin-4(3H)-one **4k**: yellow solid (0.229 g, 45% yield); ^1^H NMR (DMSO-*d*_6_, 400 MHz) : δ ppm 4.45 (ABX, 2H), 7.49-7.71 (m, 7H), 7.85-7.90 (m, 2H), 8.03 (dd, 1H, *J* = 9.0, 3.0 Hz), 8,12 (d, 1H, *J* = 9.0 Hz), 8,27 (bs, 1H) ; ^13^C NMR (DMSO-*d*_6,_ 100 MHz) : δ ppm 36.0, 117.5, 119.0, 120.6, 121.9, 122.6, 126.5, 127.0, 127.4, 128.3, 128.6, 129.0, 131.4, 132.0, 132.4, 135.0, 137.8, 142.2, 146.6, 151.8, 155.3, 161.1; HPLC, Tr = 3.32 min; MS (ESI+): calc. for C_22_H_15_N_5_O_3_S 508.0, found 508.0 [M+H]^+^, 510.0 [M+2+H]^+^.

3-(4-bromophenyl)-2-(((5-nitro-1H-benzo[*d*]imidazol-2-yl)thio)methyl)-quinazolin-4(3H)-one **4l**: brown solid (0.153 g, 30% yield); ^1^H NMR (DMSO-*d*_6_, 400 MHz) : δ ppm 4.45 (s, 2H), 7.54-7.60 (m, 4H), 7.55 (d, 1H, *J* = 9.0 Hz), 7.69-7.74 (m, 3H), 7.88 (td, 1H, *J* = 8.0, 4.0 Hz), 8.03 (dd, 1H, *J* = 8.0, 4.0 Hz), 8.14 (dd, 1H, *J* = 8.0, 4.0 Hz), 8.28 (d, 1H, *J* = 4.0 Hz); ^13^C NMR (DMSO-*d*_6_, 100 MHz) : δ ppm 36.5, 109.9, 117.2, 120.6, 126.5, 127.0 (2C), 127.3, 128.0 (2C), 129.2, 129.5, 131.1, 131.7, 133.6, 135.0, 137.8, 143.2, 146.7, 152.3, 161.1, 165.9; HPLC, Tr = 3,30 min; MS (ESI+): calc. for C_22_H_15_N_5_O_3_S 508.0, found 508.0 [M+H]^+^, 510.0 [M+2+H]^+^.

3-(3-chlorophenyl)-2-(((5-nitro-1H-benzo[*d*]imidazol-2-yl)thio)methyl)-quinazolin-4(3H)-one **4m**: yellow solid (0.158 g, 34% yield); ^1^H NMR (DMSO-*d*_6_, 400 MHz) : δ ppm 4.46 (ABX, 2H), 7.52-7.60 (m, 5H), 7.70 (d, 1H, *J* = 8.0 Hz), 7.77-7.78 (m, 1H), 7.88 (td, 1H, *J* = 8.0, 4.0 Hz), 8.03 (d, 1H, *J* = 8.0 Hz), 8.13 (dd, 1H, *J* = 8.0, 4.0 Hz), 8.27 (bs, 1H), 13.29 (bs, 1H) ; ^13^C NMR (DMSO-*d*_6_, 100 MHz) : δ ppm 36.0, 106.8, 110.7, 113.1, 117.2, 117.7, 120.6, 126.5, 127.0, 127.4, 128.0, 129.2, 129.8, 131.1, 133.6, 135.0, 137.8, 140.1, 142.7, 146.6, 151.8, 161.1; HPLC, Tr = 3.34 min; MS (ESI+): calc. for C_22_H_15_N_5_O_3_SCl 464.1, found 464,0 [M+H]^+^, 465,0 [M+2+H]^+^.

3-(4-methyl-3-nitrophenyl)-2-(((5-nitro-1H-benzo[*d*]imidazol-2-yl)thio)methyl)quinazolin-4(3H)-one **4n**: beige solid (0.332 g, 68% yield); ^1^H NMR (DMSO-*d*_6_, 300 MHz) : δ ppm 2.37 (s, 3H), 4.52 (s, 2H), 7.51-7.61 (m, 3H), 7.71 (d, 1H, *J* = 9.0 Hz), 7.84-7.92 (m, 2H), 8.03 (d, 1H, *J* = 9.0, 3.0 Hz), 8.14 (d, 1H, *J* = 6.0 Hz), 8.23 (d, 1H, *J* = 3.0 Hz), 8.31 (d, 1H, *J* = 6.0 Hz ); ^13^C NMR (DMSO-*d*_6_, 75 MHz) : δ ppm 19.1, 35.7, 117.4, 119.7, 120.6, 125.4, 126.4, 127.0 (2C), 127.4, 133.7, 134.0, 134.2, 134.9 (2C), 135.0, 142.1, 146.6, 148.9, 151.9 (2C), 155.0, 161.2 ; HPLC, Tr = 3,25 min; MS (ESI+): calc. for C_23_H_18_N_6_O_5_S 4689.1, found 489.0 [M+H]^+^.

7-methyl-2-(((5-nitro-1H-benzo[*d*]imidazol-2-yl)thio)methyl)-3-(3-(trifluoro-methyl)phenyl)quinazolin-4(3H)-one **4o**: beige solid (0.312 g, 61%); ^1^H NMR (DMSO-*d*_6_, 300 MHz) : δ ppm 2.48 (bs, 3H), 7.42 (d, 1H, J = 6.0 Hz), 7.53 (bs, 1H), 7.54 (d, 1H, *J* = 9.0 Hz), 7.79 (d, 3H, *J* = 6.0 Hz), 7.91 (d, 2H, *J* = 9.0 Hz), 8.01-8.07 (m, 4H), 8.23 (d, 1H, *J* = 3.0 Hz); ^13^C NMR (DMSO-*d*_6_, 75 MHz) : δ ppm 19.1, 35.7, 106.7, 110.6, 113.0, 117.6, 120.6, 122.3, 125.3, 126.3, 126.5, 127.0, 127.4, 130.0, 130.4, 130.8, 133.5, 135.0, 137.2, 140.2, 146.6, 151.9, 154.4, 161.2; HPLC, Tr = 3,25 min; MS (ESI+): calc. for C_24_H_17_F_3_N_5_O_3_S 512.1, found 512.0 [M+H]^+^.

7-methyl-2-(((5-nitro-1H-benzo[*d*]imidazol-2-yl)thio)methyl)-3-phenyl-quinazolin-4(3H)-one **4p**: yellow solid (0.345g, 78%); ^1^H NMR (DMSO-*d*_6_, 300 MHz) : δ ppm 2.48 (bs, 3H), 4.40 (bs, 2H), 7.39 (d, 1H, *J* = 9.0 Hz), 7.45-7.64 (m, 7H), 8.02 (d, 2H, *J* = 9.0 Hz), 8.24 (d, 1H, *J* = 3.0 Hz); ^13^C NMR (DMSO-d6, 75 MHz) : δ ppm 21.3, 36.1, 117.7, 118.2, 119.4, 124.6, 126.3, 126.5, 126.6, 127.8, 128.5, 128.8 (2C), 129.2, 129.3, 129.6 (2C), 136.5, 145.4, 146.7, 152.1, 155.8, 160.9; HPLC, Tr = 3,22 min; MS (ESI+): calc. for C_23_H_18_N_5_O_3_S 444.1, found 444.1 [M+H]^+^.

**Synthesis of quinazolines 5 :**

A solution of compound **4** (0.3 mmol) and SnCl_2_.H_2_O (1.5 mmol, 5 equiv.) in MeOH (40 mL) was refluxed for 2 h. After completion of the reaction (HPLC monitoring), the mixture was evaporated to dryness. 20 mL of dichloromethane and 20 mL of saturated aqueous NaHCO_3_ were added and the mixture was stirred for 16 h at room temperature. The mixture was then filtered through a pad of Celite and the organic layer separated. The aqueous layer was extracted with CH_2_Cl_2_ and the combined organic layer were washed with water, dried (Na_2_SO_4_), and evaporated in vacuo to give **5**.

2-(((5-amino-1H-benzo[*d*]imidazol-2-yl)thio)methyl)-3-(3-(trifluoromethyl)-phenyl)quinazolin-4(3H)-one **5a**: yellow solid (0.127 g, 91% yield); mp: 135.0-140.0°C; ^1^H NMR (CDCl_3_, 300 MHz) : δ ppm 3.98 (m, 2H), 6.58 (dd, 1H, J = 6.0, 3.0 Hz), 6.75 (bs, 1H), 7.27 (d, 1H, J = 12.0 Hz), 7.52-7.68 (m, 5H), 7.72 (t, 3H, J = 12.0 Hz), 7.81 (t, 1H, J = 8.0 Hz), 8.26 (d, 1H, J = 8.0 Hz); ^13^C NMR (CDCl_3_, 100 MHz) : δ ppm 35.9, 112.5, 119.3, 121.0, 122.0, 124.7, 126.0, 126.8, 127.7, 128.3, 131.1, 132.5, 132.7, 133.0, 135.5 (2C), 136.7, 142.7, 146.3, 146.7, 153.7, 157.7, 161.8; FT-IR: γ_max_ (cm^-1^): 3067, 2917, 1680, 1634, 1602, 1589, 1568, 1472, 1443, 1328, 1268, 1166, 1124, 1069, 805, 771, 698; HPLC, Tr = 2,44 min; MS (ESI+): m/z 468,2 [M+H]^+^; HRMS calc. for C_23_H_17_F_3_N_5_O_3_S 468.1106, found 468,1104.

2-(((5-amino-1H-benzo[*d*]imidazol-2-yl)thio)méthyl)-3-(2-(trifluorométhyl)-phényl)quinazolin-4(3H)-one **5b**: brown solid (0.074g, 53% yield); ^1^H NMR (DMSO-*d*_6_, 400 MHz) : δ ppm 4.17 (bs, 2H), 4.85 (bs, 2H), 6.42 (m, 2H), 7.07 (m, 1H), 7.60 (t, 1H, *J* = 8.0 Hz), 7.70 (d, 1H, *J* = 8.0 Hz), 7.75-7.80 (m, 1H), 7.87-7.93 (m, 3H), 8.00 (d, 1H, *J* = 8.0 Hz), 8,13 (dd, 1H, *J* = 8.0, 1.2 Hz); ^13^C NMR (DMSO-*d*_6_, 100 MHz) : δ ppm 36.1, 94.0, 110.5, 117.5, 120.0, 121.6, 124.3, 126.1, 126.4, 127.0, 127.5, 127.6, 127.7 (2C), 130.7, 131.9, 133.8, 134.2, 135.2, 144.5, 146.4, 152.1, 161.0; FT-IR: γ_max_ (cm^-1^): 3349, 3047, 2917, 1683, 1632, 1598, 1473, 1453, 1408, 1350, 1314, 1286, 1214, 1173, 1108, 1059, 1035, 806, 769, 696; HPLC, Tr = 2,26 min; MS (ESI+): m/z 468,0 [M+H]^+^; HRMS calc. for C_23_H_17_N_5_OS 468.1106, found 468,1104.

2-(((5-amino-1H-benzo[*d*]imidazol-2-yl)thio)methyl)-3-(4-(trifluoromethyl)-phenyl)quinazolin-4(3H)-one **5c**: pink solid (0.074g, 53% yield); mp: 205.8 – 208.2°C; ^1^H NMR (DMSO-*d*_6_, 400 MHz): δ ppm 4.26 (s, 2H), 6.46 (dd, 1H, J = 8.5, 1.8 Hz), 6,54 (bs, 1H), 7.06 (d, 1H, *J* = 8.5 Hz), 7.55 (t, 1H, *J* = 8.0 Hz), 7.67 (d, 1H, *J* = 8.1 Hz), 7.68-8.0 (m, 5H), 8.13 (d, 1H, *J* = 8.0 Hz); ^13^C NMR (DMSO-d6, 100 MHz): δ ppm 36.5, 96.2, 110.9, 120.5, 122.4, 125.1, 126.4, 126.5, 126.6, 126.9 (2C), 127.2, 129.5, 129.8, 130.2 (2C), 134.9, 140.2, 143.7, 144.5, 146.6, 152.0, 161.1; FT-IR : γ_max_ (cm^-1^) : 3369, 3067, 2789, 1683, 1633, 1591, 1470, 1413, 1324, 1283, 1165, 1126, 1104, 1065, 1020, 831, 696; HPLC, Tr = 2.38 min ; MS (ESI+): m/z 468.0 [M+H]+; HRMS calc. for C_23_H_17_F_3_N_5_OS 468.1106, found 468.1103.

2-(((5-amino-1H-benzo[*d*]imidazol-2-yl)thio)methyl)-3-phenylquinazolin-4(3H)-one **5d**: beige solid (0.098 g, 82% yield), mp: 132,0-134,3°C; ^1^H NMR (CDCl_3_, 300 MHz): δ ppm 4.00 (s, 2H), 6.60 (dd, 1H, J = 8.4, 2.1 Hz), 6.79 (s, 1H), 7.31-7.34 (m, 3H), 7.51-7.58 (m, 4H), 7.73 (d, 1H, *J* = 7.5 Hz), 7.83 (td, 1H, *J* = 8.4, 1.5 Hz), 8,29 (dd, 1H, *J* = 7.8, 1.2 Hz); ^13^C NMR (CDCl_3_, 75 MHz): δ ppm 36.1, 110.3, 113.4, 117.2, 120.7, 126.5, 126.9, 127.2, 128.8 (2C), 129.4, 129.6 (2C), 134.8, 136.5, 139.6, 141.9, 144.1, 146.6, 152.2, 155.9, 161.1; FT-IR: γ_max_ (cm^-1^): 3345, 3063, 2920, 1680, 1634, 1606, 1588, 1568, 1490, 1472, 1447, 1407, 1352, 1329, 1276, 1211, 1169, 1118, 1024, 994, 806, 768, 696 ; HPLC, Tr = 1,95 min; MS (ESI+) : m/z 400,1 [M+H]^+^; HRMS calc. for C_22_H_18_N_5_OS 400.1232, found 400.1231.

2-(((5-amino-1H-benzo[*d*]imidazol-2-yl)thio)methyl)-3-cyclohexylquinazolin-4(3H)-one **5e**: beige solid (0.091 g, 75% yield); mp: 131.9- 136.6°C; ^1^H NMR (DMSO-*d*_6_, 400 MHz) : δ ppm 1.11-1.82 (m, 8H), 2.56 (m, 2H), 4.23 (bs, 1H), 4.84 (bs, 3H), 6.47 (d, 1H, *J* = 8.4, 1.6 Hz), 6.59 (bs, 1H), 7.16 (d, 1H, *J* = 8.4 Hz), 7.48 (td, 1H, *J* = 8.0, 1.2 Hz), 7.55 (d, 1H, *J* = 8.0 Hz), 7.77 (td, 1H, *J* = 8.4, 1.6 Hz), 8.10 (dd, 1H, *J* = 8.0, 1.2 Hz) ; ^13^C NMR (DMSO-*d*_6_, 100 MHz) : δ ppm 24.8, 25.3 (2C), 28.0 (2C), 37.1, 59.8, 110.9, 121.5, 125.9, 126.4 (2C), 126.9, 128.3, 128.7, 134.2, 136.7, 144.4, 146.0, 153.5, 161.5; FT-IR : γ_max_ (cm^-1^): 3341, 3194, 2928, 2853, 1667, 1633, 1607, 1589, 1567, 1475, 1443, 1408, 1353, 1318, 1214, 1167, 1143, 1009, 895, 806, 770, 696; HPLC, Tr = 2.42 min; MS (ESI+): m/z 406.1 [M+H]^+^; HRMS calc. for C_22_H_24_N_5_OS 406.1702, found 406.1699.

2-(((5-amino-1H-benzo[*d*]imidazol-2-yl)thio)methyl)-3-(*m*-tolyl)quinazolin-4(3H)-one **5f**: brown solid (0.067 g, 54% yield); ^1^H NMR (DMSO-*d*_6_, 400 MHz) : δ ppm 2.34 (s, 3H), 4.23 (ABX, 2H), 6.42 (dd, 1H, *J* = 8.4, 2.0 Hz), 6.52 (bs, 1H), 7.07 (d, 1H, *J* = 8.4 Hz), 7.29-7.32 (m, 3H), 7.43 (t, 1H, *J* = 8.4 Hz), 7.56 (td, 1H, *J* = 8.0, 1.2 Hz), 7.66 (d, 1H, *J* = 8.0 Hz), 7.86 (td, 1H, *J* = 8.4, 1.6 Hz), 8.12 (dd, 1H, *J* = 8.0, 1.2 Hz) ; 13C NMR (DMSO-*d*_6_, 100 MHz) : δ ppm 20.7, 36.5, 96.7, 110.7, 120.6, 125.7, 126.3, 126.8 (2C), 127.0, 128.3, 129.1 (2C), 129.2, 129.9, 133.5, 134.7, 136.3, 139.0, 144.2, 146.6, 152.7, 161.1; FT-IR: γ_max_ (cm^-1^) : 3349, 3210, 3059, 2918, 1674, 1633, 1598, 1583, 1567, 1472, 1407, 1350, 1285, 1213, 1163, 1110, 806, 771, 694; HPLC, Tr = 2.11 min; MS (ESI+): m/z 414.0 [M+H]^+^; HRMS calc. for C_23_H_20_N_5_O_2_S 414.1389, found 414.1388.

2-(((5-amino-1H-benzo[*d*]imidazol-2-yl)thio)methyl)-3-(2-methoxyphenyl)-quinazolin-4(3H)-one **5g**: beige solid (0.080 g, 62% yield); mp: 143.6-145.6°C; ^1^H NMR (DMSO-*d*_6_, 400 MHz) : δ ppm 3.77 (s, 3H), 4.22 (ABX, 2H), 6.42 (d, 1H, *J* = 8.0 Hz), 6.52 (bs, 1H), 7,11 (dd, 2H, *J* = 15.0, 9.0 Hz), 7,24 (d, 1H, *J* = 8.0 Hz), 7.48-7.69 (m, 4H), 7.86 (t, 1H, *J* = 15.0, 9.0 Hz), 8.12 (d, 1H, *J* = 9.0 Hz); ^13^C NMR (DMSO-*d*_6_, 100 MHz): δ ppm 36.0, 54.8, 55.8, 110.7, 112,5 (2C), 116,3, 119,4, 120,4, 120,8, 124,4, 126,4 (2C), 126,9, 127,1, 130,0, 131,1, 134,8, 144,0, 146,6, 153,0, 154,3, 160,5 ; FT-IR: γ_max_ (cm^-1^) : 3349, 3063, 2920, 2853, 1678, 1632, 1606, 1569, 1498, 1472, 1438, 1408, 1353, 1332, 1278, 1251, 1212, 1162, 1125, 1110, 1044, 1021, 806, 771, 753, 697; HPLC, Tr = 2,02 min; MS (ESI+): m/z 430.1 [M+H]^+^; HRMS calc. for C_23_H_20_N_5_O_2_S 430.1338, found 430.1339.

2-(((5-amino-1H-benzo[*d*]imidazol-2-yl)thio)methyl)-3-(3-methoxyphenyl)-quinazolin-4(3H)-one **5h**: brown solid (0.082 g, 64% yield); mp: 142.3-144.9°C; ^1^H NMR (DMSO *d*_6_, 400 MHz): δ ppm 3.78 (s, 3H), 4.28 (s, 2H), 6.42 (d, 1H, *J* = 8.4 Hz), 6.52 (bs, 1H), 7.06 - 7.11 (td, 3H, *J* = 7.6, 2.4 Hz), 7.18 (bs, 1H), 7.46 (t, 1H, *J* = 8.0 Hz), 7.56 (t, 1H, *J* = 8.0 Hz), 7.66 (d, 1H, *J* = 8.0 Hz), 7.86 (t, 1H, *J* = 8.4 Hz), 8.14 (d, 1H, *J* = 8.0 Hz); ^13^C NMR (DMSO *d*_6_, 100 MHz): δ ppm 36.4, 55.3, 110.7, 114.5, 115.0, 120.6, 120.8, 126.3, 126.8 (2C), 127.0, 130.2, 132.0, 134.7 (2C), 137.5, 144.2, 146.6 (2C), 152.6, 159.9, 161.0 (2C); FT-IR: γ_max_ (cm^-1^): 3341, 3198, 2922, 1674, 1587, 1566, 1489, 1440, 1409, 1352, 1328, 1290, 1214, 1167, 1127, 1028, 803, 770, 694; HPLC, Tr = 2,08 min; MS (ESI+): m/z 430.1 [M+H]^+^; HRMS calc. for C_23_H_20_N_5_O_2_S 430,1338, found 430.1338.

2-(((5-amino-1H-benzo[*d*]imidazol-2-yl)thio)methyl)-3-(4-methoxyphényl)-quinazolin-4(3H)-one **5i**: beige solid (0,117 g, 91% yield); mp: 144.4-148.8°C; ^1^H NMR (DMSO-*d*_6_, 400 MHz): δ ppm 3.79 (s, 3H), 4.24 (bs, 2H), 6.42-6.53 (m, 2H), 7.05-7.10 (m, 3H), 7.42 (d, 2H, *J* = 8.8 Hz), 7.55 (t, 1H, *J* = 8.0 Hz), 7.65 (d, 1H, *J* = 7.6 Hz), 7,85 (t, 1H, *J* = 8.0 Hz), 8,11 (d, 1H, *J* = 8.0 Hz); ^13^C NMR (DMSO-*d*_6_, 100 MHz): δ ppm 36.7, 55.2, 94.0, 110.5, 114.4, 114.5, 117.6, 120.6, 126.2 (2C), 126.4, 126.5, 126.8, 127.0, 128.8 (2C), 129.4, 129.9, 134.6, 146.6, 153.1, 159.4, 161.3; FT-IR: γ_max_ (cm^-1^): 3357, 3067, 3000, 2915, 1679, 1633, 1608, 1592, 1469, 1411, 1353, 1299, 1278, 1248, 1167, 1106, 1029, 829, 784, 696; HPLC, Tr = 1,98 min; MS (ESI+): m/z 430,1 [M+H]^+^; HRMS calc. for C_23_H_20_N_5_OS 430.1338, found 430.1330.

2-(((5-amino-1H-benzo[*d*]imidazol-2-yl)thio)methyl)-3-(2-bromophenyl)-quinazolin-4(3H)-one **5j**: beige solid (0.071 g, 50% yield); mp: 230,5-231,6°C; ^1^H NMR (DMSO-*d*_6_, 400 MHz): δ ppm 2.08 (s, 2H), 4.19 (bs, 2H), 6.41-6.49 (m, 2H), 7.07 (bs, 1H), 7.47 (td, 1H, *J* = 7.6, 1.6 Hz), 7.58 (dd, 1H, *J* = 8.4, 1.2 Hz), 7.71 (d, 1H, *J* = 8.0 Hz), 7.76 (dd, 1H, *J* = 8.0, 1.6 Hz), 7.88-7.93 (m, 2H), 8.16 (dd, 1H, *J* = 8.0, 1.2 Hz); ^13^C NMR (DMSO-*d*_6_, 100 MHz): δ ppm 54.9, 94.0, 110.5, 117.6, 120.3, 122.4, 126.5, 127.0, 127.4, 129.1, 131.1, 131.5, 133.3, 135.1, 135.4, 135.7, 136.5, 143.8, 144.5, 146.5, 151.8, 160.2 ; FT-IR : γ_max_ (cm^-1^) : 3459, 3369, 2575, 1689, 1635, 1607, 1572, 1458, 1418, 1406, 1350, 1330, 1287, 1276, 1240, 1210, 1186, 1024, 995, 900, 825, 798, 767, 757, 718, 693; HPLC, Tr = 2.20 min; MS (ESI+) : m/z 478.0 [M+H]^+^; HRMS calc. for C_22_H_17_N_5_OSBr 478.0337, found 478.0335.

2-(((5-amino-1H-benzo[d]imidazol-2-yl)thio)methyl)-3-(3-bromophenyl)-quinazolin-4(3H)-one **5k:** beige solid (0.110 g, 77% yield); mp: 148.7-150.1°C; ^1^H NMR (DMSO-*d*_6_, 400 MHz): δ ppm 4.25 (s, 2H), 6.43 (dd, 1H, *J* = 8.4, 2.0 Hz), 6.53 (bs, 1H), 7.08 (d, 1H, *J* = 8.4 Hz), 7.50-7.72 (m, 5H), 7.85-7.90 (m, 2H), 8.12 (dd, 1H, *J* = 8.0, 1.2 Hz); ^13^C NMR (DMSO-*d*_6_, 100 MHz): δ ppm 36.4, 96.1, 110.7, 119.5, 120.5, 121.7, 126.3 (2C), 126.9, 127.1, 128.2, 131.2, 131.9 (2C), 132.3, 134.8, 137.8, 143.9, 146.6, 152.2, 158.9, 161.0; FT-IR : γ_max_ (cm^-1^): 3357, 2916, 1685, 1633, 1607, 1584, 1472, 1414, 1350, 1329, 1278, 1241, 1178, 1161, 998, 880, 799, 770, 730, 696; HPLC, Tr = 2,25 min; MS (ESI+): m/z 478.0 [M+H]^+^; HRMS calc. for C_22_H_17_N_5_OSBr 478.0337, found 478.0336.

2-(((5-amino-1H-benzo[*d*]imidazol-2-yl)thio)methyl)-3-(4-bromophenyl)-quinazolin-4(3H)-one **5l**: white solid (0.084 g, 59% yield); mp: 140,3-141,2°C; ^1^H NMR (DMSO-*d*_6_, 400 MHz): δ ppm 4.23 (s, 2H), 6.43 (dd, 1H, *J* = 12.0, 4.0 Hz), 6,53 (bs, 1H), 7,09 (d, 1H, *J* = 8.0 Hz), 7.48-7.60 (m, 3H), 7.66 (d, 1H, *J* = 7.8 Hz), 7.73-7.79 (m, 2H), 7.86 (td, 1H, *J* = 8.0, 2.0 Hz), 8.13 (dd, 1H, *J* = 8.0, 4.0 Hz); 13C NMR (DMSO-*d*_6_, 100 MHz): δ ppm 36.7, 96.9, 110.8, 117.1, 120.6, 122.7, 126.5, 127.0, 127.2, 127.9, 131.2 (2C), 132.6 (2C), 134.9, 135.9, 144.2, 146.7, 149.3, 152.4, 159.2, 161.1; FT-IR: γ_max_ (cm^-1^): 3656, 3349, 2917, 1682, 1633, 1595, 1469, 1412, 1350, 1334, 1279, 1148, 1065, 1010, 984, 818, 789, 695 ; HPLC, Tr = 2.21 min; MS (ESI+): m/z 477.9 [M+H]+; HRMS calc. for C_22_H_17_BrN_5_OS 478.0337, found 478.0340.

2-(((5-amino-1H-benzo[*d*]imidazol-2-yl)thio)methyl)-3-(3-chlorophenyl)-quinazolin-4(3H)-one 5m: beige solid (0.096 g, 74% yield), mp: 133,7-134,5°C; ^1^H NMR (DMSO-*d*_6_, 400 MHz): δ ppm 4.26 (s, 2H), 6.46 (dd, 1H, *J* = 8.0, 4.0 Hz), 6.57 (bs, 1H), 7.09 (d, 1H, *J* = 8.0 Hz), 7.54-7.59 (m, 5H), 7.66 (t, 1H, *J* = 8.0 Hz), 7.75 (bs, 1H), 7.87 (td, 1H, *J* = 8.0, 4.0 Hz), 8.12 (d, 1H, *J* = 8.0 Hz); 13C NMR (DMSO-*d*_6_, 100 MHz) : δ ppm 36.5, 94.5, 99.5, 109.8, 109.9, 111.1, 120.6, 126.5, 127.0, 127.3, 127.9, 129.2, 131.0, 131.1, 133.4, 133.6, 137.8, 137.9, 143.3, 146.7, 152.3, 161.1; FT-IR : γ_max_ (cm^-1^): 3357, 3057, 2922, 1679, 1633, 1607, 1585, 1472, 1414, 1354, 1330, 1282, 1212, 1175, 993, 775, 746, 696; HPLC, Tr = 2.17 min; MS (ESI+): m/z 434.1 [M+H]^+^; HRMS calc. for C_22_H_17_N_5_OSCl 434.0842, found 434.0843.

2-(((5-amino-1H-benzo[*d*]imidazol-2-yl)thio)methyl)-3-(3-amino-4-methyl-phenyl)quinazolin-4(3H)-one **5n**: brown solid (0.081 g, 63% yield); mp: 164,9-165,9°C; ^1^H NMR (CD_3_OD, 400 MHz): δ ppm 2.07 (s, 3H), 2.17-2.30 (m, 1H), 4.21 (s, 2H), 6.52 (d, 1H, *J* = 8.0 Hz), 6.65 (s, 1H), 6.72 (d, 1H, *J* = 8.4 Hz), 6.80 (s, 1H), 6.98 (d, 1H, *J* = 8.0 Hz), 7.21 (d, 1H, *J* = 8.4 Hz), 7.48-7.54 (m, 2H), 7.77 (t, 1H, *J* = 8.4 Hz), 8.17 (d, 1H, *J* = 8.0 Hz) ; ^13^C NMR (CD_3_OD, 100 MHz): δ ppm 17.4, 54.9, 100.2, 114.7, 115.4, 116.7, 118.2, 122.1, 125.3, 128.2 (2C), 129.4 (2C), 135.6 (2C), 140.3, 144.4, 147.3 (2C), 148.5, 155.1, 164.1; FT-IR : γ_max_ (cm^-1^): 3338, 3206, 2922, 2853, 1670, 1629, 1588, 1566, 1503, 1472, 1407, 1352, 1296, 1210, 1113, 994, 800, 770, 694; HPLC, Tr = 1.76 min; MS (ESI+): m/z 429.1 [M+H]^+^; HRMS calc. for C_23_H_21_N_6_OS 429.1498, found 429.1500.

2-(((5-amino-1H-benzo[*d*]imidazol-2-yl)thio)methyl)-7-methyl-3-(3-(trifluoro-méthyl)phenyl)quinazolin-4(3H)-one **5o**: beige solid (0.093 g, 64.5% yield); mp: 143.7- 144.9°C; ^1^H NMR (CDCl_3_, 300 MHz): δ ppm 2.52 (s, 3H), 4.03 (s, 2H), 6.56 (d, 1H, *J* = 9.0 Hz), 6.74 (s, 1H), 7.24-7.72 (m, 9H), 8.10 (d, 1H, *J* = 9.0 Hz) ; ^13^C NMR (CDCl_3_, 100 MHz): δ ppm 22.3, 36.0, 112.5, 118.5, 122.1, 124.8, 126.1, 126.1, 126.6, 127.0, 127,1, 127,5, 129.8, 131.1, 132.6, 132.7, 133.1, 136.8, 142.7, 146.5, 146.8 (2CH), 153.8, 161.8; FT-IR : γ_max_ (cm^-1^) : 3345, 3067, 2919, 2849, 1680, 1631, 1606, 1590, 1567, 1497, 1441, 1409, 1353, 1328, 1290, 1165, 1125, 1069, 1022, 828, 803, 784, 755, 698; HPLC, Tr = 2,59 min; MS (ESI+): m/z 482.0 [M+H]^+^; HRMS calc. for C_24_H_19_F_3_N_5_OS 482.1262, found 482.1261.

2-(((5-amino-1H-benzo[*d*]imidazol-2-yl)thio)methyl)-7-methyl-3-phenylquinazolin-4(3H)-one **5p**: beige solid (0.102 g, 82% yield); mp: 156.7-157.5°C; ^1^H NMR (CDCl_3_, 300 MHz): δ ppm 2.55 (s, 3H), 3.98 (s, 2H), 6.58 (d, 1H, *J* = 9.0 Hz), 6.80 (s, 1H), 7.30-7.54 (m, 8H), 8.16 (d, 1H, *J* = 9.0 Hz); 13C NMR (CDCl_3_, 100 MHz) : δ ppm 22.1, 36.0, 112.5, 118.5, 118.8, 126.3, 127.3, 127.6, 128.7, 129.4, 129.6, 129.9, 130.2, 130.5, 136.3, 142.6, 146.5, 147.7, 154.8, 155.2, 161.9; FT-IR: γ_max_ (cm^-1^): 3218, 3035, 2918, 1676, 1615, 1589, 1562, 1509, 1454, 1335, 1252, 1233, 1175, 1141, 1020, 810, 761, 729, 695; HPLC, Tr = 2.75 min; MS (ESI+): m/z 414.2 [M+H]^+^; HRMS calc. for C_23_H_20_N_5_OS 414.1310, found 414.1312.
